# CDCA3 Predicts Poor Prognosis and Affects CD8^+^ T Cell Infiltration in Renal Cell Carcinoma

**DOI:** 10.1155/2022/6343760

**Published:** 2022-09-28

**Authors:** Yuanyuan Bai, Shangfan Liao, Zhenjie Yin, Bingyong You, Dongming Lu, Yongmei Chen, Daoxun Chen, Yongyang Wu

**Affiliations:** Department of Urology, Affiliated Sanming First Hospital, Fujian Medical University, Sanming, 365100 Fujian, China

## Abstract

**Background:**

Cell division cycle associated 3 (CDCA3) mediates the ubiquitination WEE1 kinase at G2/M phase. However, its contribution to cancer immunity remains uncertain.

**Methods:**

We first evaluated the effect of CDCA3 on the prognosis of patients with renal cell carcinoma (RCC). The results of bioinformatics analysis were verified by the tissue microarray, immunofluorescence (IF) staining, CCK-8 assay, colony formation, cell cycle, and Western blot.

**Results:**

Bioinformatics analysis predicated CDCA3 was an independent predictor of poor prognosis in RCC and was associated with poor TNM stage and grade. CDCA3 was related to the infiltration of CD8^+^ T cells and Tregs. Tissue microarray demonstrated that CDCA3 was strongly associated with poor prognosis and positively relevant to CD8^+^ T infiltration. In vitro experiments showed that exgenomic interference of CDCA3 could attenuate cellular proliferation, arrest cell cycle, and blockade accumulation of CDK4, Bub3, and Cdc20 in mitosis process.

**Conclusion:**

CDCA3 presents as a good biomarker candidate to predict the prognosis of RCC patients and potentiates the immune tumor microenvironment (TME) of RCC.

## 1. Introduction

Renal cell carcinoma (RCC) is a malignancy from the kidney epithelium and the mobility has steadily increased globally in recent years [1]. The first-line antiangiogenic therapies such as tyrosine kinase inhibitors (TKI) have presented the certain effect for RCC patients, however, the response is discontinued in short time for the majorities [2]. Immune checkpoint inhibitors (ICIs) usher a new time of cancer therapeutic strategies via sparking anticancer immunity [3]. CD8^+^ T cells serve as an essential effector and partially relevant to the effect of ICI [4]. Traditionally, RCC is considered as an immunogenic cancer, and immunotherapy has shown a certain effect of RCC [5, 6]. In clinical practices, we observe the effect of ICIs is diversified, however, scholars fail to find a good candidate to predicate the response and adverse effects (AEs) of ICI in RCC treatment. The biomarkers will also help identify subgroups that respond to immunotherapy and avoid severe AEs.

Cell division malfunctions trigger tumor development and antitumor immune response [7]. CDCA3 has been shown to be a poor prognostic factor for renal papillary cell carcinoma, nonsmall cell lung cancer, etc. [8–10]. Scholars reveal that CDCA3 was upregulated in RCC and promote tumor progression and sunitinib resistance [11] via activating the NF-*κ*B/cyclin D1 signaling axis [12]. There are data indicating that CDCA3 can serve as an important biomarker to evaluate the therapeutic sensitivity of TKI and therefore it would be appropriate to underline this aspect also in light of the possible associations of immunological therapies and TKI in various types of malignant tumors [13]. Moreover it should be very interesting to test the role of CDCA3 as a predictive biomarker of toxicity related to a prolonged use of these novel agents in combination therapy of RCC [14]. However, the immune impact of CDCA3 has also not been well reported.

In this paper, we try to evaluate the predicable performance of CDCA3 in RCC and figure out the attribution of CDCA3 to TME of RCC. Finally, we endorse that targeting CDCA3 would be a potential therapeutic way to flight RCC.

## 2. Methods and Materials

### 2.1. Data Collection and Preprocessing

The RNA-seq data, clinical information, somatic mutation data, and microsatellite instability (MSI) status of 881 RCC were all from The Cancer Genome Atlas (TCGA, https://portal.gdc.cancer.gov/) portal. Patients were divided into high expression group and low expression group based on the median of gene expression. We first drew Kaplan-Meier (KM) survival curve, receiver operating characteristic (ROC) curve, and risk curve to study the prognosis of patients in terms of overall survival (OS). Next, we analyzed the differences in clinical data including gender, clinical stage, TNM stage, and grade among different expression groups of CDCA3. In addition, we used univariate and multivariate COX regression to analyze the prognostic significance of CDCA3 expression and clinical data. At the same time, we drew a nomogram diagram and calibration curve to better interpret the prognostic significance of CDCA3. Moreover, fold change = 2 was used to compare the differences of gene expression among different expression groups of CDCA3, and a heat map of differentially expressed genes was drawn to show the expression trend in different groups. Finally, considering that CDCA3 can be used as an oncogene to affect the progression of tumor, we performed Gene Ontology (GO) and Kyoto Encyclopedia of Genes and Genomes (KEGG) enrichment analysis on the upregulated genes of CDCA3 in different expression groups to identify CDCA3 functional pathway localization in tumors.

### 2.2. Correlation between Tumor Immune Cell Infiltration and CDCA3 Gene Expression

Cell type Identification By Estimating Relative Subsets Of RNA Transcripts (CIBERSORT) algorithm was used to estimate the infiltration proportion of 22 kinds of immune cells in normal kidney and RCC samples to describe the profile of immune cell infiltration in RCC. The abundance of immune cells infiltration and the expression of 8 important immune checkpoints (CD274, CTLA4, HAVCR2, LAG3, PDCD1, PDCD1LG2, TIGIT, and SIGLEC15) among different CDCA3 expression groups were compared. Finally, we also analyzed the correlation between CDCA3 expression with tumor mutation burden (TMB) and MSI. Tumor Immune Single-cell Hub (TISCH, http://tisch.comp-genomics.org/) is a scRNA-seq database focusing on TME. We obtained the relationship between CDCA3 and RCC TME at single-cell level in TISCH.

### 2.3. Cell Culture and Transfection of Lentivirus

Caki-1 and 786-O were purchased from the Type Culture Collection (Chinese Academy of Sciences, Shanghai, China). Cells were cultured in RPMI-1640 medium (HyClone, USA) with 10% fetal bovine serum (Gibco, Grand Island, NY, USA). The culture was maintained in a humidified incubator with 37°C, 5% CO_2_. CDCA3 knockdown lentivirus was designed by Obio Technology Corp (Shanghai, China). Then, Caki-1 and 786-O were transfected with the lentivirus, according to the manufacturer's instructions. Two days later, puromycin was added for screening. Knockdown efficiencies of CDCA8 were assessed by Western blot.

### 2.4. Western Blotting

Cultured cell lysates were prepared using a Column Tissue & Cell Protein Extraction Kit (Epizyme, Shanghai, China; # PC201PLUS). Then total proteins were then separated on 10% SDS polyacrylamide gels. After overnight incubation with various primary antibodies, including anti-CDCA3 (Proteintech, 15594-1-AP), CDK4 (Proteintech, 11026-1-AP), Cdc20 (Proteintech, 10252-1-AP), Bub3 (Proteintech, 27073-1-AP), and anti-GADPH (CST, #5174) at 4°C, membranes were washed thrice for 5 min each time, using TBST (in 0.1% Tween20). Then, they were incubated in the presence of a secondary rabbit antibody (1 : 1000, LF102, Epizyme) for 1 h and washed thrice using TBST for 5 min each time. Signals were detected using the chemiluminescence system.

### 2.5. Cell Proliferation Assay

The cells were seeded in 96-well plates (1,000 cells/well) and cultured for 1, 2, and 3 days. After adding 10 *μ*l CCK-8 (Dojindo, Japan) to each well and incubating at 37°C for 2 h, the absorbance at 450 nm was measured by the Rayto-6000 system (Rayto, China).

### 2.6. Colony Formation Assay

For cell proliferation, we seeded 200 cells to each well of 6-well plates for 14 days, then fixed with 4% paraformaldehyde (PFA) and stained with crystal violet. The cells were photographed, and the numbers of colonies were counted.

### 2.7. Flow Cytometry

Cell cycle analysis was performed using a Cell Cycle Staining Kit (MultiSciences, Hangzhou, China), as instructed by the manufacturer. Cells were washed using PBS, after which 1 ml of DNA staining solution and 10 *μ*l of permeate were added to the cell suspension and vortexed to mix. Finally, cells were stained in the dark at 4°C for 30 min and analyzed by flow cytometry. The stained cells were assessed by flow cytometry (BD FACSCanto [TM] II, USA), and analysed by FlowJo vX.0.7 software.

### 2.8. Tissue Microarray

The RCC tissue microarray was purchased from Outdo (Shanghai, China) and contains 150 RCC tissues and 30 paired paracancer tissues along with their survival, clinical information, etc. Samples were collected from the National Human Genetic Resources Sharing Service platform (2005DKA21300). All points on the chip were detected by Immunofluorescence (IF). The expression of CDCA3, CD8, CD4, CD68, FOXP3, and PD-1 was detected by intensity and positive number of IF. We divided 150 RCC patients into two groups based on the optimal CDCA3 cut-off value and plotted survival curves to identify their prognostic significance. Finally, we analyzed the correlation between CDCA3 and CD8, CD4, FOXP3, CD68, and PD-1.

### 2.9. Immunofluorescence Staining

Tissue microarray were deparaffinized by graded alcohol and then washed three times with phosphate-buffered saline (PBS), permeabilized with 0.4% Triton X-100 for 30 min, and blocked with goat serum working liquid (Wuhan Boster Biological Technology, Wuhan, China) for 2 hours after antigen retrieval. The sections were then incubated overnight with mixed primary antibodies at 4°C, washed in PBS to remove unbound primary antibodies, and incubated with secondary antibodies in the dark at room temperature (RT) for 1 hour. The sections were counterstained with 4′, 6 diamidino-2-phenylindole (Sigma-Aldrich) for 5 minutes and washed with PBS. The primary antibodies included CDCA3 (Proteintech, 15594-1-AP). The fluorophore-conjugated secondary antibodies used were goat anti-rabbit Alexa Fluor 488 (1: 500; Abbkine, Wuhan, China) and goat anti-mouse Alexa Fluor 549 (1: 500; Abbkine, Wuhan, China). Images were captured by confocal laser scanning microscopy (Nikon A1 + R, Japan). The fluorescence intensity was analyzed by using the ImageJ software.

### 2.10. Statistical Analysis

In this study, R (version 4.0.2), GraphPad Prism 8, and SPSS 20.0 software were used to analyze the data. Survival, survminer, timeROC, rms, Limma, ggplot2, pheatmap, and ClusterProfiler R package were used in this study. The significance of differences between groups was assessed by the student *T* test. Chi-square test was used for categorical variables, and Wilcoxon test was used for continuous data. Survival differences were calculated using Kaplan-Meier and logarithmic rank tests.

## 3. Results

### 3.1. Prognostic Significance of CDCA3 in RCC

**Figure 1:**
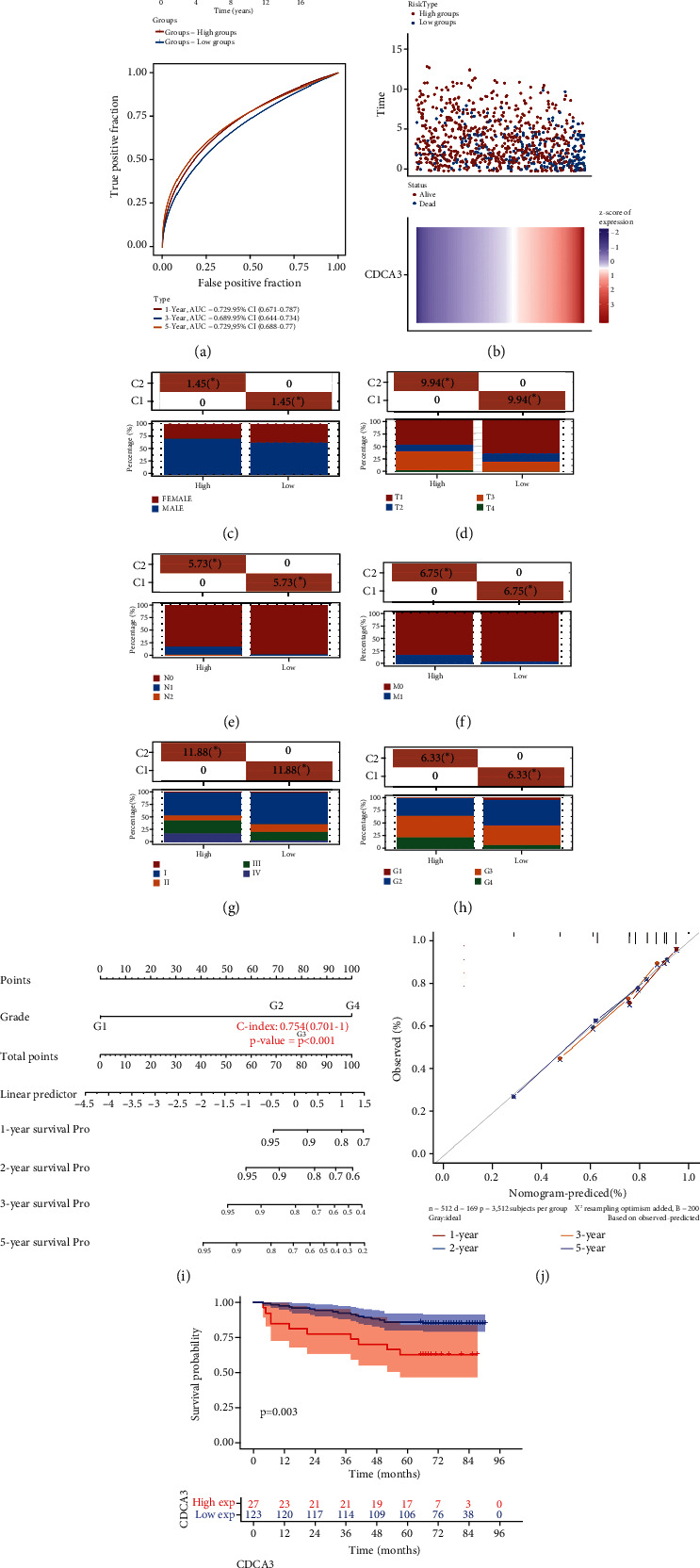
CDCA3 may be an independent prognostic factor for RCC. Survival curves, ROC curves (a), and mortality risk curves (b) of patients with different CDCA3 expression levels. (c–h) Comparison of distribution of gender, T, N, M stage, clinical stage, and grade among patients with different CDCA3 expression. CDCA3-related nomogram (i) and calibration curve (j) predict OS of the RCC patients. (k) Survival curve of patients with different CDCA3 expression in tissue microarray.(^∗^*p* < 0.05, ^∗∗^*p* < 0.01, ^∗∗∗^*p* < 0.001).

First, KM survival analysis of TCGA-RCC revealed a shorter survival time in the high-CDCA3 expression group versus the low-CDCA3 expression group (*p* < 0.001, *n* = 881). ROC curves suggested a good accuracy of CDCA3 expression in predicting RCC prognosis (*AUC* = 0.729, Figure 1(a)). The risk curve showed higher mortality in high-CDCA3 patients than low-CDCA3 patients (Figure 1(b)). Among the patients with different CDCA3 expression groups, gender, TNM stage, clinical stage, and grade showed differences in distribution (Figures 1(c)–1(h)). Univariate and multivariate COX analysis showed that CDCA3, age, TNM stage, and grade could be used as prognostic factors of RCC, and CDCA3 could independently predict the prognosis of RCC (Table 1). We also constructed the prognostic nomogram and calibration curve of RCC, and the 5-year overall survival rate could be estimated according to the total score (C − index = 0.754, Figures 1(i) and 1(j)). Demographic characteristics and pathological baseline of tissue microarray were listed in Table 2, showing that high CDCA3 expression levels predicted shorter survival (*p* = 0.003, Figure 1(k)), which proves the bioinformatics analysis. In summary, CDCA3 can be an independent prognostic factor and reflect the rate of tumor progression tumor progression in RCC.

### 3.2. CDCA3 Is Related to Immune Infiltration

**Figure 2:**
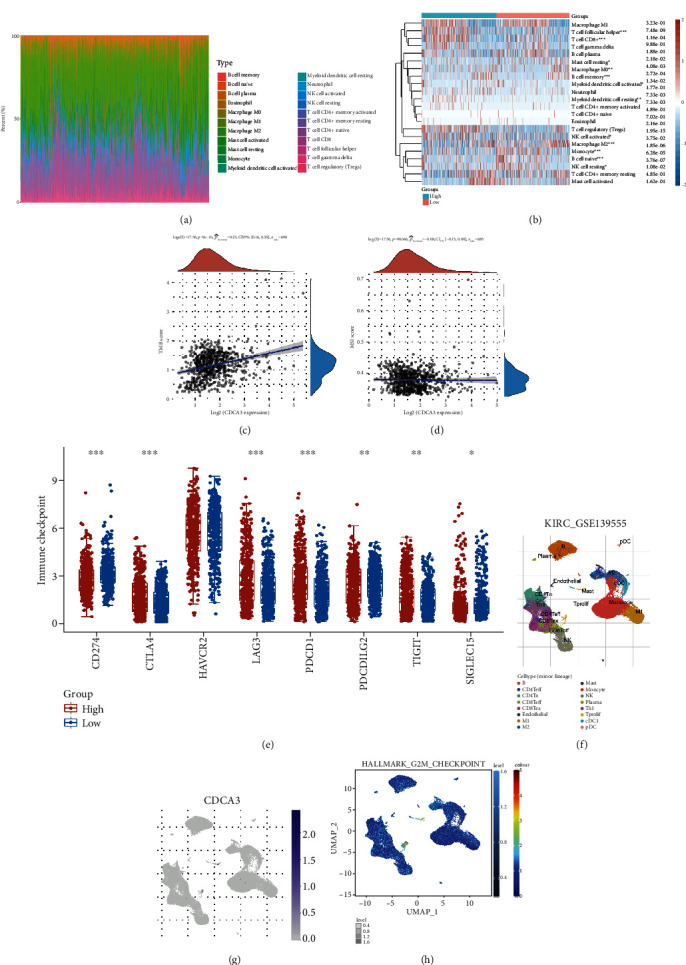
CDCA3 affects immune infiltration in RCC. (a) The distribution of immune cells infiltration in RCC. (b) Comparison of immune cells infiltration between different CDCA3 expression groups. (c) Correlation analysis between CDCA3 and TMB. (d) Correlation analysis between CDCA3 expression and MSI. (e) Comparison of 8 immune checkpoints in different expression groups of CDCA3. (f) Single-cell level distribution of immune cells in RCC. (g) Expression of CDCA3 in immune cells. (h) The relationship between CDCA3 and the G2/M checkpoint in immune cells. (^∗^*p* < 0.05, ^∗∗^*p* < 0.01, ^∗∗∗^*p* < 0.001).

Figure 2(a) showed the infiltration of immune cells in RCC. On this basis, we further analyzed the different abundance of immune cell infiltration among different CDCA3 expression groups (Figure 2(b)). The infiltration of CD8^+^ T cell (*p* < 0.001), Tregs (*p* < 0.001), memory B cell (*p* < 0.001), follicular helper T cell (*p* < 0.001), activated NK cell (*p* < 0.05), and M0 macrophage (*p* < 0.01) was upregulated in the patients with high expression of CDCA3, while naive B cell (*p* < 0.001), resting NK cell (*p* < 0.05), Monocyte (*p* < 0.001), and M2 macrophage (*p* < 0.001) was downregulated. TMB and MSI levels reflect tumor surface neoantigen abundance and can stimulate antitumor immune response. CDCA3 was also positively correlated with TMB (*p* < 0.001, *r* = 0.23, Figure 2(c)) and negatively correlated with MSI (*p* = 0.046, *r* = −0.08, Figure 2(d)). CD274 (PD-L1, *p* < 0.001), PDCD1LG2 (PD-L2, *p* < 0.01), and SIGLEC15 (*p* < 0.05) were downregulated in patients with high expression of CDCA3, while CTLA4 (*p* < 0.001), LAG3 (*p* < 0.001), PDCD1 (PD-1, *p* < 0.001), and TIGIT (*p* < 0.01) were upregulated (Figure 2(e)). The distribution of immune cells in KIRC is shown in Figure 2(f).  Figure 2(g) shows immune cells hardly express CDCA3. CDCA3 can regulate G2/M phase, so we analyzed the relationship between CDCA3 and immune cells G2/M checkpoint. Our results show a broad association of CDCA3 with immune cell G2/M checkpoints (Figure 2(h)).

**Figure 3:**
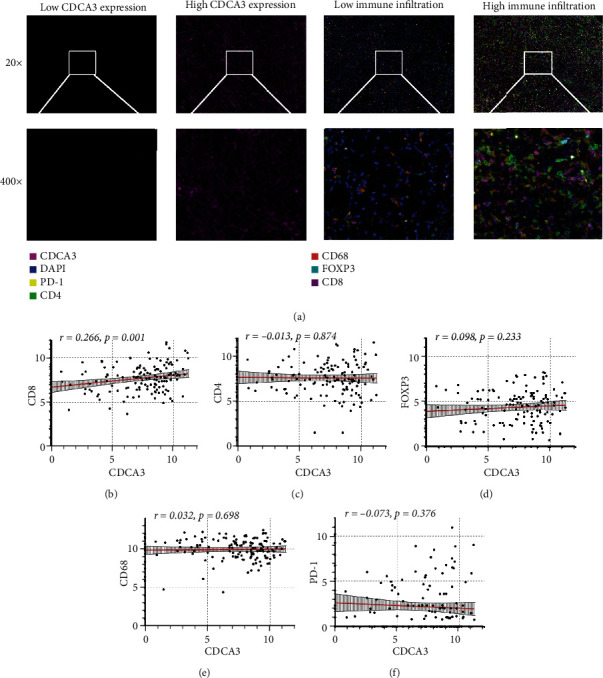
The relationship between CDCA3 and immune cell infiltration in tissue microarray. (a) Representative images of IF staining in tissues microarray. (b–f) Correlation between CDCA3 and CD8, CD4, FOXP3, CD68, FOXP3, and PD-1.

Further, we conducted tissue microarray to try to prove the above results.  Figure 3(a) shows that we performed IF staining in RCC tissue microarray. There was a significant positive correlation between CDCA3 and CD8 (Figure 3(b)). However, our study did not observe the correlation between CDCA3 and CD4, FOXP3, CD68, and PD-1 (Figures 3(c)–3(f)). In conclusion, CDCA3 was closely related to tumor immune cells infiltration and antitumor immunity. And CDCA3 may be important for RCC risk stratification and immunotherapy guidance.

### 3.3. Identification of Molecular Mechanism of CDCA3

**Figure 4:**
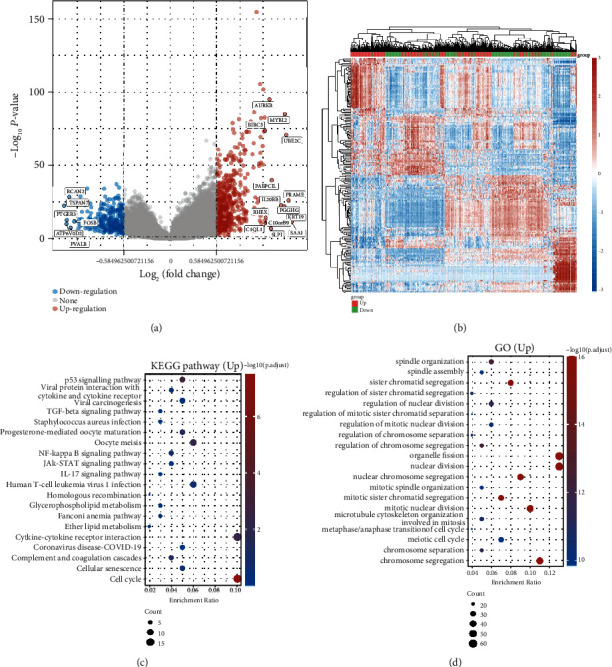
Enrichment analysis of CDCA3 positively correlated genes. (a) Distribution of differential genes with different CDCA3 expression levels. (b) The heat map of the differential genes expression in CDCA3 high and low expression groups. KEGG enrichment analysis (c) and GO enrichment analysis (d) of differential genes in CDCA3 high and low expression groups.

The distribution of different genes among patients with different CDCA3 expression groups was shown in the volcano map (Figure 4(a)). The heat map showed the expression trend of 50 upregulated genes and 50 downregulated genes with the greatest difference (Figure 4(b)). KEGG enrichment analysis showed that the related pathways were mainly concentrated in p53 signal pathway, TGF-*β* signal pathway, NF-*κ*B signal pathway, and JAK-STAT signal pathway. (Figure 4(c)). GO enrichment analysis showed that its biological function was mainly enriched in spindle organization, regulation of sister chromatid segregation, and nuclear division (Figure 4(d)). These results suggest that CDCA3 mainly affects cell cycle in RCC and may regulate antitumor immune response through NF-*κ*B axis and other important immune-related pathways.

### 3.4. CDCA3 Knockdown Attenuated RCC Cell Proliferation and Arrested Cell Cycle

**Figure 5:**
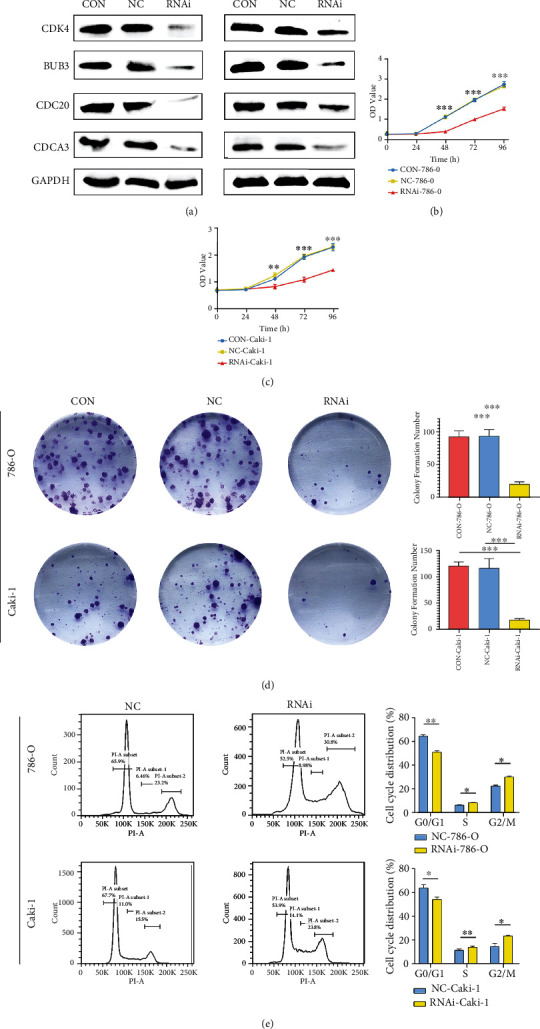
The function of CDCA3 was confirmed by in vitro experiments. (a) Western blot was used to detect the expression of CDCA3, CDK4, Bub3, and Cdc20 in different groups of cells. CCK-8 array to detect (b) 786-O and (c) Caki-1 proliferation. (d) Representative images of crystal violet stain on day 15. (e) Representative images of flow cytometry.(^∗^*p* < 0.05, ^∗∗^*p* < 0.01, ^∗∗∗^*p* < 0.001).

To further understand the effect of CDCA3 on the biological behavior of RCC, we constructed CDCA3-knockdown cell lines for functional experiments. Lentiviruses carrying CDCA3 shRNA were used to obtain CDCA3-knockdown Caki-1 and 786-O. The Western blot results showed that the expression of CDCA3 was significantly decreased in the RNAi group, indicating that the CDCA3-knockdown cell lines were successfully constructed (Figure 5(a)). Meanwhile, the expression of CDK4, BUB3, and Cdc20 was decreased (Figure 5(a)), which indicating cell cycle arrest. The CCK-8 assay showed that CDCA3 knockdown remarkably attenuated the cell proliferation (Figures 5(b) and 5(c)). The ability of colony formation was notably impaired after knockdown of CDCA3 gene (Figure 5(d)). The flow cytometric indicated that CDCA3 knockdown cause G1, S, and G2/M phase arrest (Figure 5(e)). In general, CDCA3 expression affects cell cycle operation and cell proliferation.

## 4. Discussion

Our study suggested that CDCA3 can independently predict prognosis and affect tumor progression in RCC. CDCA3 may also be involved in the regulation of immune-related pathways, and stimulated the infiltration of immune cells, such as CD8^+^ T cells and Tregs. Importantly, we verified our results in vitro.

Scholars revealed that CDCA3 influence many tumor progression and treatment through a variety of pathways and is associated with poorer prognosis [8, 15]. Our results also showed consistency. Patients with high CDCA3 expression had significantly worse survival and clinical stage, which was confirmed by our results and public databases. One important reason is that dysregulation of cell cycle is the basis of abnormal proliferation of tumor cells. We also confirmed that downregulation of CDCA3 blocked the G2/M phase of cells and reduced cell proliferation ability. This directly proved that the functional localization of CDCA3 was a key regulatory protein in the cell cycle, and its abnormal expression can affect tumor progression and prognosis.

Infiltrating immune cells directly affect the occurrence, development, and treatment of tumors. It has been reported that CDCA3 is closely related to immune infiltration in hepatocellular carcinoma [16]. Our results showed that CDCA3 affected tumor infiltration of various immune cells, including CD8^+^ T cells in endogenous and exogenous data. Previous studies have shown that CD8^+^ T cells can recognize tumor-specific antigens and played a role in tumor control [17]. The high density of tumor infiltrating CD8^+^ T cells has been proved to be associated with a good prognosis of most cancers [18], but the infiltration of CD8^+^ T cells in RCC was associated with a poor prognosis [19], this is consistent with our survival outcomes. Since immune cells hardly express CDCA3, antitumor therapy targeting CDCA3 may not cause damage to immune cells, which is a potential treatment. Our study was firstly proved that CDCA3 may be involved in the regulation of immune cell infiltration and tumorigenesis in RCC. But more importantly, the specific pathway through which CDCA3 affects immune infiltration needs further study.

As we know, immune checkpoint is a key molecule in tumor immune escape pathway. There were a lot of evidences showed that immune checkpoints were related to the benefit degree of ICIs treatment, which can be used as biomarkers for ICIs treatment [20–22]. Our results showed that patients with high expression of CDCA3 also expressed high levels of CTLA4 and PD-1. This initially showed that there was a close relationship between CDCA3 and immune checkpoints and further suggested that CDCA3 may participate in the immune pathway of RCC by regulating immune regulatory factors, which may be a potential target for immunotherapy. Moreover, findings suggested that TMB may predict clinical response to ICIs [23]. The neoantigen produced by TMB may be an important reason for stimulating antitumor response. In our study, we found that there was a positive correlation between CDCA3 and TMB, also suggesting that CDCA3 may predict the benefits of immunotherapy.

In summary, reactive TME is the key to immunotherapy, and CDCA3 helps to evaluate this phenomenon. Furthermore, enrichment analysis was performed to evaluate the actual molecular mechanism of CDCA3 in RCC. CDCA3 has been suggested to influence the NF-*κ*B pathway to mediate tumor progression [12]. Our results supported this point. CDCA3 is also involved in P53 and TGF pathways. NF-*κ*B is involved in the regulation of inflammation and innate immunity in tumor development. P53 also plays an important role in immune system. P53 mutation in cancer triggers B cell antibody response and CD8^+^ killing T cell response [24]. TGF-*β* can inhibit the proliferation, activation, and effector function of T cells. In addition, TGF-*β* further enhances immunosuppression in TME by promoting Tregs differentiation and destroying T cell immunity [25]. These evidences suggest that CDCA3 has a reasonable influence on TME, but more specific studies are needed to uncover the regulatory mechanisms.

TME is recognized as a complex dynamic ecosystem, which is composed of malignant tumor cells, various infiltration immune cells, fibroblasts, and a variety of cytokines. In this ecosystem, immune response plays an important role in tumorigenesis and development. RCC has always been regarded as an immunogenic malignant tumor [26–28], and it is usually insensitive to chemotherapy and radiotherapy. Immunotherapy is regarded as another therapeutic target in addition to chemotherapy and radiotherapy [29]. Clinicians are focusing on immunotherapy to create a new era of RCC treatment, trying to break through the traditional barrier [30]. The first thing to use immunotherapy is to evaluate the immune status, which is the premise of personalized treatment. Therefore, find a biomarker that can better indicate the immune status and curative effect of patients, which provides an important reference for immunotherapy of RCC. When we focus on CDCA3, the problem seems to become transparent. CDCA3 has the potential to evaluate prognosis and TME and helps to hierarchically label patients at high risk. Then apply medical intervention in advance, select appropriate treatment strategies, and improve the prognosis. However, our study has its limitations. First, the specific mechanism of CDCA3 on CD8^+^T cells and its influence on immunotherapy of renal cell carcinoma need to be further explored; second, we have proved that CDCA3 can block the cell cycle, but there is no further study on the biological mechanism.

## 5. Conclusion

CDCA3 can be used as an oncogene to affect the prognosis of RCC patients. Downregulation of CDCA3 causes cell stagnation in G2/M phase, promotes cell apoptosis, and reduces proliferation ability. More importantly, the immunological implications of CDCA3 have also been preliminarily evaluated. CDCA3 may participate in the regulation of immune infiltration in tumor microenvironment by affecting the expression of many immune regulatory factors and TMB, which is expected to provide valuable reference for clinical ICIs treatment. Overall, CDCA3 can be used as a biomarker to evaluate prognosis and CD8^+^ T cell infiltration in RCC. Targeted therapy against CDCA3 is a promising new therapeutic modality, and focusing on it may help to improve the management of therapeutic resistance in the combination of ICI and TKI, but this needs further research to confirm.

## Figures and Tables

**Table 1:** Univariate and multivariate COX regression analysis.

Uni-COX	*p* value	Hazard ratio (95% CI)	Multi-COX	*p* value	Hazard ratio (95% CI)
CDCA3	< 0.0001	2.34698 (2.0353, 2.70639)	CDCA3	< 0.0001	1.71293 (1.41056, 2.08011)
Age	< 0.0001	1.028 (1.01694, 1.03918)	Age	< 0.0001	1.03039 (1.01629, 1.04468)
Gender	0.4682	0.9037 (0.68738, 1.1881)			
Race	0.5979	1.10877 (0.75541, 1.62743)			
Clinical stage	< 0.0001	2.01609 (1.79567, 2.26356)	Clinical stage	< 0.0001	1.52842 (1.31228, 1.78016)
Grade	< 0.0001	2.29073 (1.86981, 2.80639)	Grade	0.0029	1.41708 (1.12665, 1.78237)

**Table 2:** CDCA3 expression and demographic and clinicopathological characteristics.

	CDCA3		*N*	*p* value
	Low	High		
Age				
≥57	58	16	74	0.666
<57	65	11	76	
Gender				
Female	33	10	43	0.408
Male	90	17	107	
Size(cm^3^)				
≤175	62	13	75	1.000
>175	61	14	75	
T				
T1-2	116	23	139	0.215
T3	7	4	11	
N				
N0	121	26	147	1.000
N1-2	2	1	3	

## Data Availability

All public data access addresses are visible in the manuscript. Data archiving will be made available on reasonable request and all of the authors are responsible to the data.
